# Microbiome Alteration in Type 2 Diabetes Mellitus Model of Zebrafish

**DOI:** 10.1038/s41598-018-37242-x

**Published:** 2019-01-29

**Authors:** Fumiyoshi Okazaki, Liqing Zang, Hiroko Nakayama, Zhen Chen, Zi-Jun Gao, Hitoshi Chiba, Shu-Ping Hui, Takahiko Aoki, Norihiro Nishimura, Yasuhito Shimada

**Affiliations:** 1 Department of Life Sciences, Graduate School of Bioresources, Mie University, 1577 Kurimamachiya, Tsu, Mie 514-8507 Japan; 2 Department of Bioinformatics, Mie University Advanced Science Research Promotion Center, Tsu, Mie Japan; 3 Mie University Zebrafish Drug Screening Center, Tsu, Mie Japan; 4 Graduate School of Regional Innovation Studies, Mie University, Tsu, Mie Japan; 5 Faculty of Health Sciences, Hokkaido University, Kita-12, Nishi-5, Kita-ku, Sapporo, 060-0812 Japan; 6 Department of Nutrition, Sapporo University of Health Sciences, Nakanuma Nishi-4-2-1-15, Higashi-ku, Sapporo 007-0894 Japan; 7 Department of Integrative Pharmacology, Mie University Graduate School of Medicine, Tsu, Mie Japan

## Abstract

Understanding the gut microbiota in metabolic disorders, including type 2 diabetes mellitus (T2DM), is now gaining importance due to its potential role in disease risk and progression. We previously established a zebrafish model of T2DM, which shows glucose intolerance with insulin resistance and responds to anti-diabetic drugs. In this study, we analysed the gut microbiota of T2DM zebrafish by deep sequencing the 16S rRNA V3-V4 hypervariable regions, and imputed a functional profile using predictive metagenomic tools. While control and T2DM zebrafish were fed with the same kind of feed, the gut microbiota in T2DM group was less diverse than that of the control. Predictive metagenomics profiling using PICRUSt revealed functional alternation of the KEGG pathways in T2DM zebrafish. Several amino acid metabolism pathways (arginine, proline, and phenylalanine) were downregulated in the T2DM group, similar to what has been previously reported in humans. In summary, we profiled the gut microbiome in T2DM zebrafish, which revealed functional similarities in gut bacterial environments between these zebrafish and T2DM affected humans. T2DM zebrafish can become an alternative model organism to study host-bacterial interactions in human obesity and related diseases.

## Introduction

Worldwide prevalence of obesity continues to increase at an alarming rate, and more than 415 million people were estimated to be suffering from type 2 diabetes mellitus (T2DM) in 2015^1^. T2DM is a metabolic disorder, which is primarily caused by obesity-induced insulin resistance, and more than 90% of people with T2DM are overweight or obese. In addition, obesity and T2DM are associated with specific changes in the gut microbiota composition. For example, many studies have found that T2DM patients have a reduced abundance of butyrate-producing species, leading to a low-grade inflammation in the gut^2^. This has been reported in people of different races, ethnicities and after controlling for the effect of anti-diabetic drugs on the gut microbiome^3^.

Over these recent decades, Zebrafish has emerged as a pre-eminent vertebrate model organism for biomedical research. The number of microbiota related studies in zebrafish are still limited but growing, in developmental and physiological microbiology^4–8^, colitis models^9–11^, effects of antibiotics^12^ and immune responses^13–17^. Among the studies on zebrafish microbiota in obesity, Semova I, *et al*. were the first reported that diet-induced alteration of gut microbiota composition influenced fat absorption^18^. After that, Felcinelli S, *et al*. demonstrated that *Lactobacillus rhamnosus*, a probiotic bacterium beneficial to humans, could modulate gut microbiota composition in zebrafish^19^ and attenuate obese phenotypes^20^. However, there are no reports that have evaluated the possibility of using zebrafish gut microbiota as a model for obesity and related metabolic disorders.

We previously created a diet-induced T2DM model of zebrafish (T2DM zebrafish), which shows glucose intolerance and insulin resistance with common obese phenotypes (body weight increase, visceral adiposity, hepatic steatosis and dyslipidaemia)^21^. This zebrafish model also shares common pathological transcriptome pathways associated with human T2DM, and shows improvement with anti-diabetic medications (metformin and glibenclamide). We believe that this model could be suitable for therapeutic target identification and chemical screening. To further evaluate the possibility of utilizing T2DM zebrafish as a model for human gut microbiota in T2DM, we conducted deep sequencing of 16S rRNA and compared T2DM-induced microbiota alternations between zebrafish and humans. Furthermore, we predicted the metabolic pathways associated with the microbiota of T2DM zebrafish using Phylogenetic Investigation of Communities by Reconstruction of Unobserved States (PICRUSt)^22^, based on the 16S rRNA sequence datasets.

## Methods

### T2DM zebrafish

All zebrafish procedures were approved by the Ethics Committee of Mie University and were performed according to the Japanese animal welfare regulation ‘Act on Welfare and Management of Animals’ (Ministry of the Environment, Japan) and complying with international guidelines. Zebrafish (AB strain; the Zebrafish International Resource Center, Eugene, OR, USA) were maintained in our facility according to standard guidelines. Each adult male zebrafish (6 months old) was assigned to either overfeed or a control group, under a single fish per 2L tank condition. To induce T2DM, zebrafish were overfed with Otohime B2 for 4 weeks, based on our previous study^21^. Body metrics (body weight, total length, standard length and height at anterior of anal fin [HAA], and body mass index [BMI])^23,24^, and fasting blood glucose^25,26^ were measured at the end of the feeding experiment.

### Gut bacterial DNA isolation

Zebrafish were sacrificed 2 h after the feeding experiment, with ice water. Each fish was then surface sterilized using 70% ethanol, and the intestinal contents were collected by gently squeezing the intestine with forceps. DNA was isolated with the PowerSoil DNA Isolation Kit (MoBio Laboratories, Carlsbad, CA, USA) according to the manufacturer’s instructions.

### 16S rRNA sequencing of intestinal content

Illumina MiSeq paired-end sequencing of the hypervariable V3-V4 regions of the 16S rRNA was performed at Bioengineering Lab. Co., Ltd., Kanagawa, Japan. A two-step, tailed PCR approach was used according to the protocol for 16S metagenomic sequencing library preparation (Illumina, San Diego, CA, USA). Both the V3 and V4 regions of the 16S ribosomal RNA were amplified with primers containing the Illumina overhang adaptor (Forward primer 5′ ACA CTC TTT CCC TAC ACG ACG CTC TTC CGA TCT CCT ACG GGN GGC WGC AG; Reverse primer 5′ GTG ACT GGA GTT CAG ACG TGT GCT CTT CCG ATC TGA CTA CHV GGG TAT CTA ATC C). Index PCR was performed with Index 1 and Index 2 Primers from the Nextera XT Index Kit (Illumina), using 2 μL of amplicon derived from the previous PCR. The indexed libraries were cleaned and analysed with Bioanalyzer, using a high sensitivity DNA kit (Agilent Technologies, Wilmington, DE). The prepared libraries were used for paired-end sequencing using MiSeq v3 reagents and 2 × 300-bp reads on the MiSeq (Illumina).

### Determination of colony forming units in the tank water

Heterotrophic bacteria in tank water were analysed by spread plating method on R2A-agar (Difco Laboratories, Sparks, MD, USA). Briefly, R2A-agar plates were inoculated with 0.1 ml of the samples and 0.1 ml of the 10^−1^ dilutions in triplicates, and spread with a sterile glass rod. All plates were incubated for 14 days at 25 °C to reach a plateau, and then the numbers of colonies were counted.

### Analysis of bacterial composition in 16S rRNA datasets

The paired-end reads of the 16S rRNA amplicons were assembled using QIIME (version 1.9.1)^27^, with the script multiple_join_paired_ends.py. Further processing of paired-end reads, including quality filtering based on a quality score of >19, removal of mismatched barcodes and sequences below length thresholds (130 bases), was performed. Quality filtered reads were assigned to Operational Taxonomic Units (OTUs) (97% identity) using *de novo* OTU picking and taxonomic assignment using the UCLUST algorithm^28^ against the Greengenes (version 13.8)^29^ reference using the script pick_de_novo_otus.py.

### Measurement of fructose and BCAA

Twelve hours after starvation, zebrafish blood was collected as described in our previous study^25,30^. Contents of the gut were collected and homogenized in D-PBS at five-times the original volume. Then, the supernatants were obtained by centrifugation at 15,000 *g* for 10 min at 4 °C. Plasma and gut fructose levels were quantified using PicoProbe Fructose Fluorometric Assay Kit (Biovision, Mountain View, CA, USA), according to manufacturer’s instruction. Branched chain amino acids were quantified using Branched Chain Amino Acid Assay Kit (Abcam, Cambridge, MA, USA), according to manufacturer’s instruction.

### Measurement of butyrate

Samples for LC-MS/MS analysis were prepared using a previously reported method^31^. In brief, 10 µL of sample containing 2.0 nmol heptanoic acid as internal standard was saponified by mixing with 25 µL of 0.3 M KOH-EtOH and heating at 80 °C for 45 min. Then, derivatization was performed by adding 100 µL 2-NPH·HCl and EDC·HCl, and incubating at 60 °C for 20 min. Fatty acid NPH derivatives were obtained using potassium phosphate buffer and diethyl ether extraction, followed by vacuum drying of the diethyl ether layer. The dried residue was dissolved in 100 µL of methanol before injection. LC-MS/MS analysis was performed using a Surveyor HPLC system and a TSQ Quantum Access MAX mass spectrometer with a heated electrospray ionization (H-ESI) probe (Thermo Fisher Scientific, Waltham, MA, USA). LC was carried out on an Ascentis Express Phenyl-Hexyl column (5 cm × 2.1 mm I.D., 2.7 µm; Supelco, Bellefonte, PA, USA) at 45 °C. The injection volume was set at 5.0 µL. The mobile phase consisted of 5 mM aqueous ammonium acetate (A), isopropanol (B), and methanol (C) at a flow rate of 200 µL/min. The following gradient elution was applied: 0.0−0.5 min 65% A and 35% C; 0.5–1.0 min 30% A, 20% B, 50% C; 1.0−4.5 min 5% A, 30% B, 65% C; 4.5–5.0 min 33% B, 67% C; this ratio was kept up to 8.0 min; 8.0–10.0 min, ratios were returned to initial gradient for re-equilibration. The selected reaction monitoring (SRM) under negative mode was utilized for MS detection, and the main parameters were optimized. Spray voltage was set at 3000 V. Nitrogen was used as the sheath gas and the auxiliary gas (set at 50 psi and 10 psi, respectively). The vaporizer temperature and the capillary temperature was set at 350 °C and 200 °C, respectively. Collision gas (argon) pressure was set at 1.8 mTorr. Quantification was performed using Thermo Xcalibur 2.1 software (Thermo Fisher Scientific).

### Diversity analysis

Diversity analyses were performed on QIIME using the default parameters. α-diversity (microbial diversity within samples) using Chao1 (a richness estimator based on OTU frequency counts) was analysed based on sample sizes, normalized using the minimum number of sequences obtained among samples. β-diversity (microbial diversity between samples) across the samples, was calculated based on unweighted UniFrac^32^ distance matrices using the script beta_diversity_through_plots.py. A Principal Coordinate Analysis (PCoA) plot was constructed showing the overall dissimilarity of bacterial communities in different groups.

### Metabolic reconstruction of 16S rRNA datasets

Phylogenetic Investigation of Communities by Reconstruction of Unobserved States (PICRUSt)^22^ was used to predict the functional gene content in the faecal microbiota based on the taxonomy obtained from the Greengenes reference database (http://greengenes.lbl.gov/cgi-bin/nph-index.cgi)^29^.

### Statistical analysis

Comparison of OTU percentages between groups was performed using the Mann-Whitney U-test, adjusted for multiple comparisons, using GraphPad Prism version 7 (GraphPad Software, San Diego, CA, USA). Data from the two groups (body metrics and plasma or faecal parameters) were analysed using a non-parametric two-sample t test. All error bars indicate SEM.

## Results and Discussion

### Hyperglycaemic-zebrafish show different composition of gut microbiota

**Figure 1:**
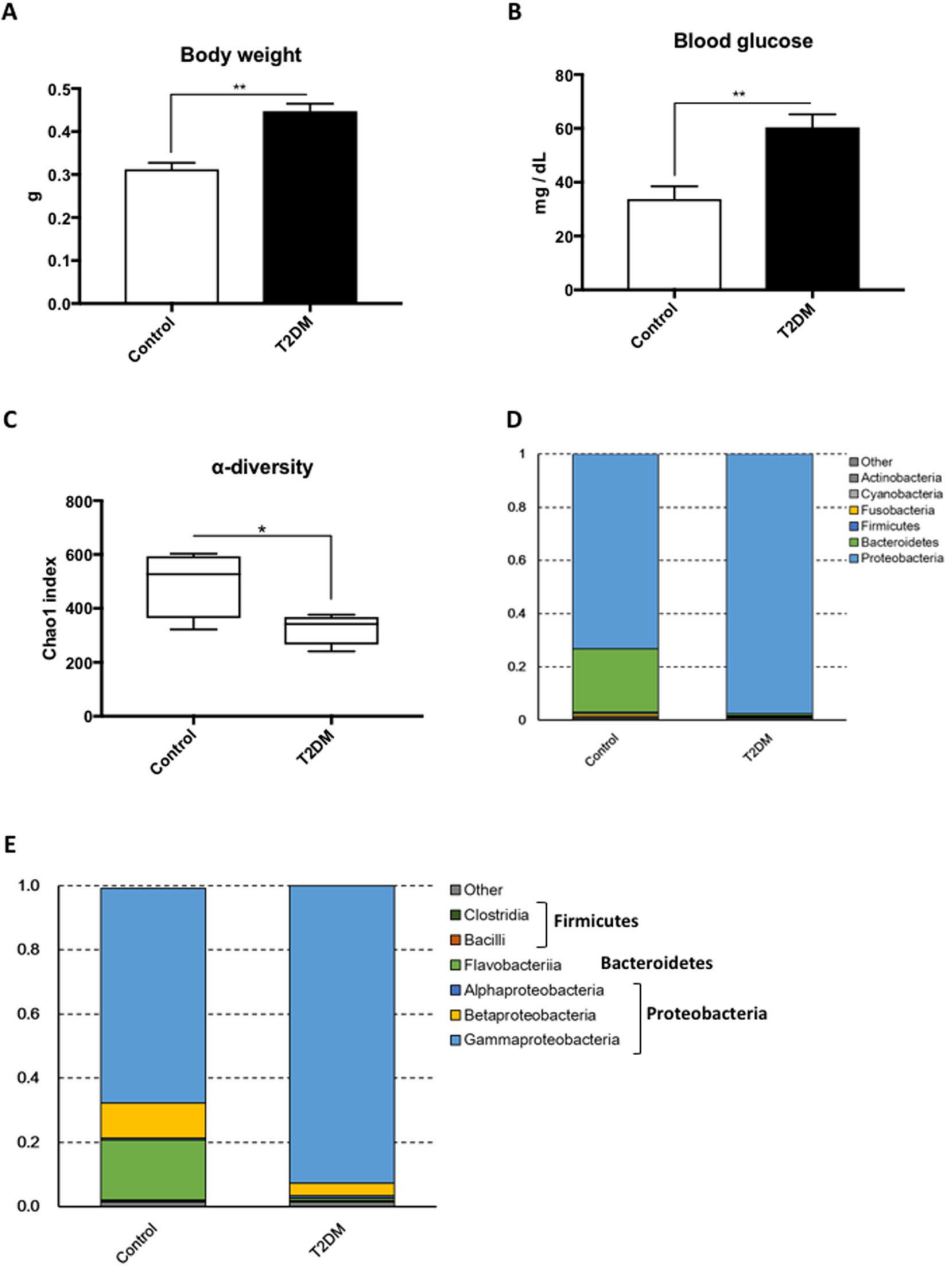
Microbiome in T2DM zebrafish. (**A**) Body weight gain after 4-weeks of feeding experiment. The T2DM (overfed) group had increased body weight compared to the control group. **p < 0.01, n = 10. (**B**) Fasting blood glucose (FBG) after the 4-week feeding experiment. The T2DM group exhibited higher FBG compared to the control group. **p < 0.01, n = 5. (**C**) α-diversity analysis. Whiskers in the boxplot represent minimum and maximum α-diversity values within each group. **p < 0.01, n = 4. (**D**,**E**) Median relative abundance of dominant bacteria at phylum level (**D**) and class level (**E**).

We created T2DM zebrafish by overfeeding them for 4-weeks (about 408 calories per fish per day) as previously described^21^. This resulted in a significant (*p* < 0.01) increase in body weight (Fig. 1A; 0.31 ± 0.02 g in controls vs. 0.45 ± 0.02 g in T2DM), HAA (Table S1; 5.50 ± 0.19 mm in controls vs. 6.60 ± 0.22 mm in T2DM) and BMI (Table S1; 0.28 ± 0.01 kg/m^2^ in controls vs. 0.34 ± 0.01 kg/m^2^ in T2DM), as a consequence of diet-induced obesity. Controls were fed 68 calories per fish per day as per normal feeding procedures. The fasting blood glucose was doubled (*p* < 0.01) in the T2DM group compared with the controls (Fig. 1B; 33.8 ± 4.7 mg/dL in control vs. 60.4 ± 4.9 mg/dL in T2DM group), consistent to our previous study^21^. We then constructed and sequenced 16S rRNA amplicon libraries from the intestinal content samples. The numbers of read tags in each sample were almost the same at about 120-thousand (Fig. S1), corresponded to 1,226 predicted species of the microbiota. Principle coordinate analysis (PCoA; Fig. S2) and jackknifed tree constructed (Fig. S3) with unweighted UniFrac^32^ revealed that bacterial composition in T2DM zebrafish was different from that of the control group. The dynamic alteration in bacterial compositions between control and T2DM zebrafish indicates that overfeeding affected the microbiota in the zebrafish gut, even though these fish were fed with the same kind of fish feed (the feeding volume of the T2DM group was five-times that of the controls). Bacterial load in the breeding water of the overfed group doubled to that of the control group after feeding experiments (Fig. S4). Because the UV-irradiated breeding water of these tanks was obtained from the same source, the environmental bacterial burden, which was induced by overfeeding and overfed-zebrafish, could not be eliminated by the regular water exchange rate (2.8 ± 0.1 min/exchange). In addition, the zebrafish fed on their faeces, which resulted in enhanced alternation of their gut microbiota in this study. The α-diversity estimates are methods used for describing the number of types of organisms in a single sample, and are useful for examining patterns of dominance, rarity, and community complexity. We evaluated the α-diversity by using the Chao1 index and found that the T2DM group showed lower (*p* < 0.05) α-diversities (Taxa richness) of operational taxonomic units (OTUs) compared to those of the controls (Fig. 1C). This result indicates that overfeeding reduced bacterial diversity in the zebrafish gut. Because a lower bacterial diversity has also been described in association with obesity and insulin resistance in human studies^33,34^, the T2DM zebrafish shares common microbiota responses with human T2DM patients in their disease state.

### Comparison of gut microbiota alteration between zebrafish and human T2DM patients

*Proteobacteria* phylum dominated the zebrafish gut microbiome (Figs 1D and S5), consistent with a previous study^5^. The second most dominant phylum was *Bacteroidetes*, which showed a tendency (*p* < 0.2) to reduce in abundance in T2DM zebrafish. Gaulke CA, *et al*. previously reported that the main phyla in adult zebrafish gut were *Proteobacteria* and *Fusobacteria*^12^, and there were very few *Bacteroidetes*. Our result was slightly different from their result. This may be attributed to the difference in feeding, Gaulke CA, *et al*. fed their zebrafish with processed fish feed containing algae as an omnivorous diet, while we mainly fed our zebrafish *Artemia* and not algae-containing processed feed. As bacterial communities are known to be different in different fish facilities^5^, we hypothesized that the difference in feed may have resulted in the difference in compositions of *Bacteroidetes* and *Fusobacteria*. In addition, *Bacteroidetes* are widely distributed in sea water, and our fish feed was made of krill and sea fish, which could be sources of *Bacteroidetes* in our zebrafish gut. Common zebrafish gut bacteria, *Cetobacterium* sp., was detected in our control group and its percentage of total reads (0.9%) is similar to the previous study^5^. Interestingly, *Cetobacterium* disappeared from the T2DM group, probably due to the reduction of bacterial diversity (Fig. 1C).

**Figure 2:**
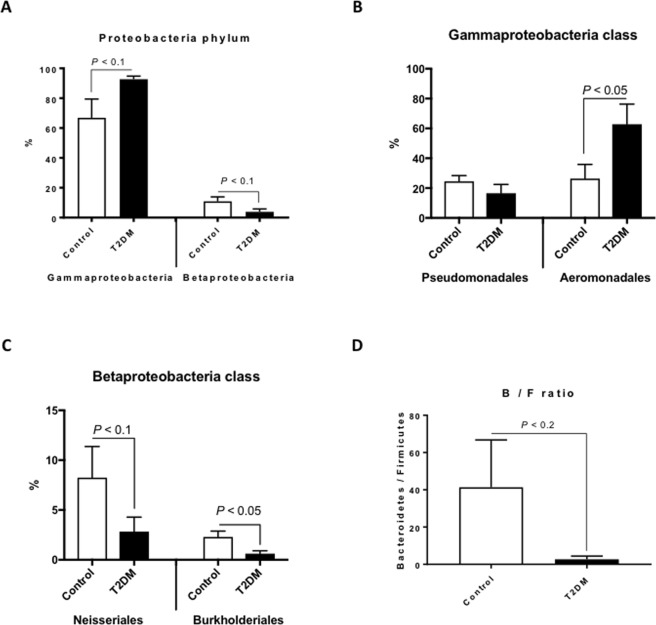
Difference in bacterial compositions between T2DM zebrafish and control. (**A**) In the *Proteobacteria* phylum, *Gamma-proteobacteria* and *Beta-proteobacteria* were increased and decreased in T2DM zebrafish compared with the control group, respectively n = 4. (**B**) In the *Gamma-proteobacteria* class, *Aeromonadales* were increased in T2DM zebrafish compared to the control group n = 4. (**C**) In the *Beta-proteobacteria* class, *Neisseriales* and *Burkholderiales* were decreased in T2DM zebrafish compared to the control group n = 4. (**D**) The *Bacteroidetes*-to-*Firmicutes* (B/F) ratio in T2DM and control zebrafish. n = 4.

At the class level, bacterial compositions between controls and T2DM zebrafish was also different (Figs 1E and S6). *Gamma-proteobacteria*, *Beta-proteobacteria* and *Flavobacteriia* dominated in the control zebrafish. *Gamma-proteobacteria* class was slightly increased (*p* < 0.1) and the *Beta-proteobacteria* class was slightly decreased (*p* < 0.1) in T2DM zebrafish (Fig. 2A). In the *Gamma-proteobacteria* class, order *Aeromonadales* was significantly (*p* < 0.05) increased in T2DM (26.3% in controls vs. 62.8% in T2DM; Fig. 2B). The majority of *Aeromonadales* belonged to the *Aeromonadaceae* family, as reported in a previous zebrafish study^35^, and *Aeromonadaceae* was also increased in human T2DM patients^36,37^, consistent with the result of our T2DM zebrafish. In addition, even while occurring in a small proportions in the microbiome (less than 2%), *Alteromonadales* and *Vibrionales* orders in the *Gamma-proteobacteria* class were significantly (*p* < 0.01 and *p* < 0.05, respectively) reduced in T2DM compared to the controls (Fig. S7), reflecting the reduction of α-diversity in T2DM zebrafish (Fig. 1C). In the *Beta-proteobacteria* class, orders *Neisseriales* and *Burkholderiales* were decreased (*p* < 0.1 and *p* < 0.05, respectively) in T2DM (Fig. 2C). *Burkholderiales* included *Oxalobacter* and related genera, which is unusual since they use oxalic acid as their carbon source in the human colon, to prevent the formation of kidney stones^38^. Because kidney stone formation, T2DM and obesity are all highly related to the gut microbiome^39^, our result indicates a possibility that reduction *in Burkholderiales* may promote kidney stone formation in T2DM zebrafish and humans.

Several studies in mice and humans have provided evidence that an increase in body weight is associated with a larger proportion of *Firmicutes* and fewer *Bacteroidetes*^33,40,41^. Even though *Firmicutes* were not the main bacteria in our zebrafish, the *Bacteroidetes*-to-*Firmicutes* ratio in T2DM zebrafish tended (*p* < 0.2) to be lower than that of the control group (Fig. 2D). This was caused by the decrease of the *Bacteroidetes* phylum (*p* < 0.2, Fig. 1D), mainly the *Flavobacteriia* class of the *Bacteroidetes* phylum (18.7% in controls vs. 0.7% in T2DM; *p* < 0.1, Fig. S8). Because *Firmicutes* generate more harvestable energy than *Bacteroidetes*, it seems reasonable that obese humans and rodents have relatively more *Firmicutes*^42^. However, we did not detect many *Firmicutes* in the zebrafish gut (less than 1%), which implies that other phyla compensate for the decrease of *Bacteroidetes* to produce enough energy. In fact, Semova I *et al*. reported that *Firmicutes* were rich (about 30%) only in zebrafish embryos (6 day-post-fertilization), and not in adults (less than 1%)^18^, suggesting that other phyla compensate for the lack of *Firmicutes* in adult fish. *Akkermansia muciniphila*, which improves insulin resistance in humans^43,44^, was not detected in our zebrafish.

### Metabolic functional pathways in T2DM zebrafish microbiome

**Figure 3:**
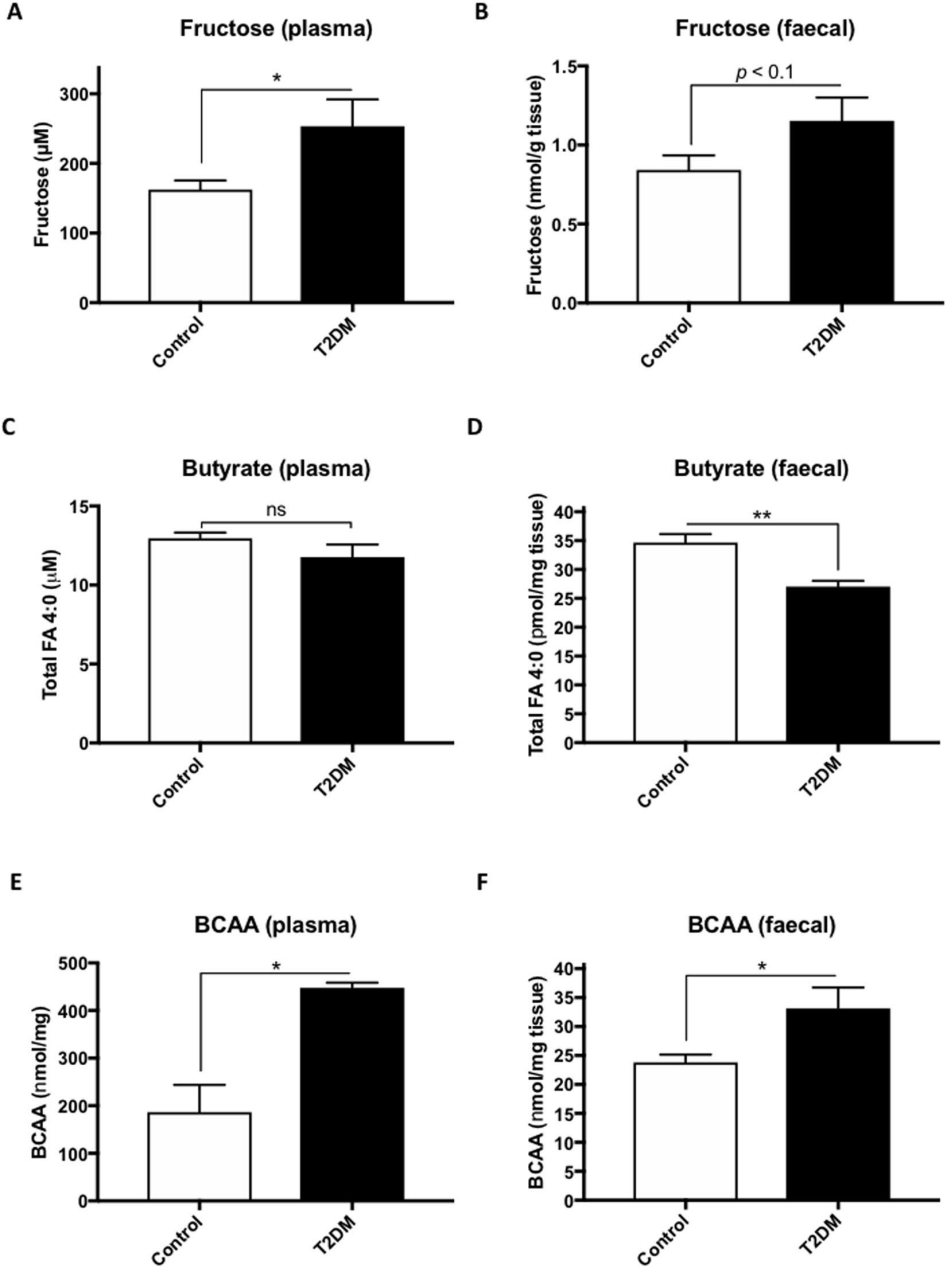
Alteration of microbial KEGG metabolic pathways in T2DM zebrafish. (**A**–**C**) Several KEGG metabolic pathways were downregulated in the microbiome of T2DM zebrafish compared with those of the control group. Arginine and proline metabolism (**A**), phenylalanine metabolism (**B**) and butyrate (butanoate) metabolism (**C**). Red and green indicates downregulation and upregulation, respectively. KEGG pathway maps (ko00330, ko00360, ko00650) are adapted here from http://www.kegg.jp/kegg/kegg1.html. The KEGG database has been described previously^46^.

To understand the T2DM-induced metabolic alterations in gut microbiota, bacterial metagenomes were predicted by PICRUSt using 16S rRNA sequencing as previously reported^45^. Predicted proteins in each bacterium were classified into KEGG ortholog entities, which resulted in the identification of 6909 entities across all samples. Of these, we selected 965 ortholog entities that were increased (>2) or decreased (<0.5) in T2DM zebrafish compared to the control group, and used them to construct pathways using KEGG Mapper^46^. Our analysis revealed 55 reference pathways that contained more than five ortholog entities each (Table S2**)**. Apart from Glycolysis/Gluconeogenesis (KO00010, contains 10 entities) and the Citrate cycle pathway (KO00020, contains nine entities), several pathways of amino acid metabolisms are listed below. The pathway with the most entities, amino sugar and nucleotide sugar metabolism (KO00529, contains 26 entities), is particularly well-studied in bacteria because these molecules are required for the synthesis of glycoconjugates on the surfaces of these organisms^47^. The dysregulation of this pathway is caused by hyperglycaemia in T2DM zebrafish because of the excessive sugars inside and/or outside the gut epithelium. In addition, the fructose and mannose metabolism (KO00051, contains 17 entities) pathway were also dysregulated in T2DM zebrafish (Fig. S9), consistent with a human T2DM study^48^. Fructose is closely related to glucose, with growing evidence of its contribution to several metabolic disorders, including obesity and T2DM^49,50^. Excessive fructose in the gut results in bacterial fermentation, which leads to formation of short-chain fatty acids (SCFAs)^51^. Several types of SCFAs have been reported to improve T2DM features^52^, while the end products were associated with arteriosclerosis and colon cancer^53^. Thus, the health promoting function of SCFAs can be still disputed and needs to be reconsidered. We also focused on the metabolic pathways of arginine and proline (KO00330, contains 22 entities; Fig. 3A) and phenylalanine (KO00360, contains 20 entities; Fig. 3B) in T2DM zebrafish. Downregulation of arginine and proline metabolism (Fig. 3A; red entities) results in the decrease of glutamic acid via a reduction of glutamate-5-semialdehyde. Arginine and its metabolites promote insulin secretion^54,55^ and improve insulin resistance in an obese mice^56,57^ and humans^58^. Regarding phenylalanine (Fig. 3B), various metabolome analysis revealed that its levels are altered in blood^59^, plasma^60^, blood cells, and urine^61^ in human T2DM. The pathway for butyrate (butanoate) metabolism (KO00650, 17 entities), a downstream pathway of arginine and proline metabolism, was also downregulated (Fig. 3C).

**Figure 4:**
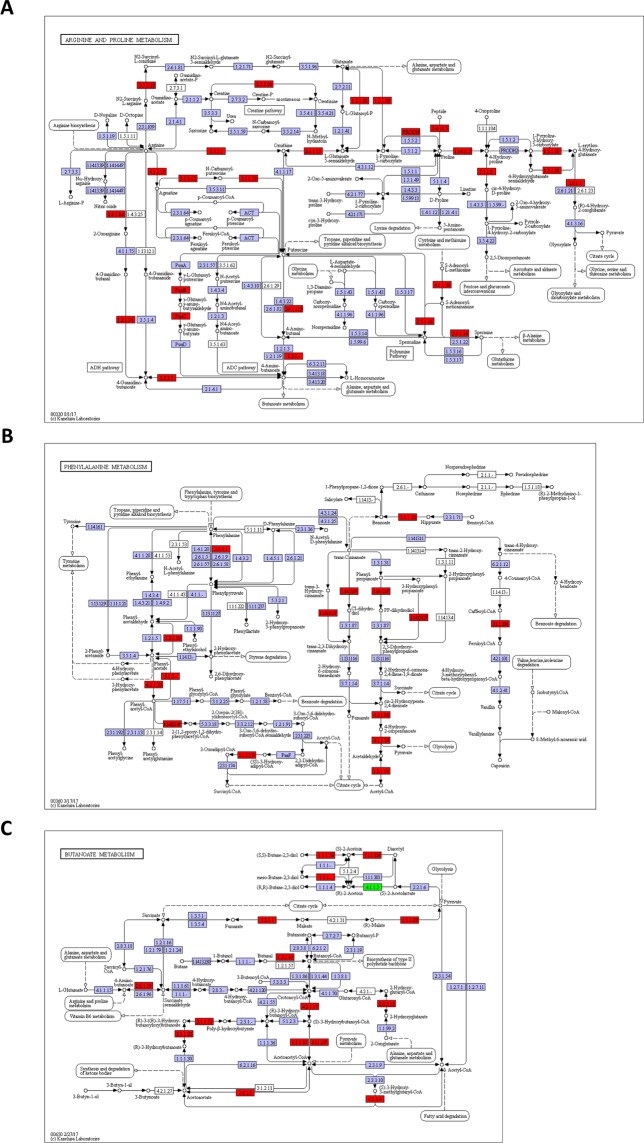
Alteration of fructose, butyrate and BCAA in plasma and faeces of T2DM zebrafish. (**A**,**B**) Plasma (**A**) and faecal (**B**) fructose levels in zebrafish. (**C**,**D**) Plasma (**C**) and faecal (**D**) butyrate levels in zebrafish. (**E**,**F**) Plasma (**E**) and faecal (**F**) branched-chain amino acids (BCAA) in zebrafish. *p < 0.05, **p < 0.01, n = 4–5.

To validate the predicted functional content of the microbiome in T2DM zebrafish, we measured plasma and faecal levels of fructose. Both plasma and faecal fructose levels increased in T2DM zebrafish compared to the controls (Fig. 4A,B), in accordance with the switch from fructose to mannose metabolism (Fig. S9), similar to that in rodents and humans^62,63^. We also measured the plasma and faecal concentration of butyrate (Total FA 4:0). As shown in Fig. 4C,D, only faecal butyrate levels were significantly (*p* < 0.05) reduced in T2DM zebrafish compared to the controls, in accordance with the downregulation of butyrate metabolism pathway (Fig. 3C). Dietary supplementation of butyrate improves insulin sensitivity and increases energy expenditure in mice^64^. In addition, butyrate producing intestinal bacteria seems to play an important role in blood glucose regulation and lipid metabolism, as shown by faecal transplantation studies in humans^65,66^. Because branch-chain amino acids (BCAA) have been shown to be associated with insulin resistance and T2DM in humans^67,68^, we also measured plasma and faecal BCAA in the zebrafish. Similar to T2DM studies in humans, T2DM zebrafish showed higher BCAA concentration in their plasma (Fig. 4E) and faeces (Fig. 4F). Although faecal transplantation in zebrafish has not been reported yet, probably because of the lack of germ-free adult zebrafish^69^, we expect normal microbiota transfer into T2DM zebrafish gut to improve these dysregulations and insulin resistance. Our results indicate that microbiota in T2DM zebrafish adversely affects its health via inhibition of these amino acid metabolisms.

## Summary

In this study, we performed 16S rRNA sequencing and functional metagenomics profiling of T2DM zebrafish gut microbiome. The main class in the normal zebrafish gut was *Gamma-proteobacteria*, *Beta-proteobacteria* and *Flavobacteriia*. In addition, *Beta-proteobacteria* and *Flavobacteriia* were decreased in T2DM. As for bacterial metabolic functions predicted using PICRUSt and KEGG, several pathways of amino acid metabolism were dysregulated in T2DM, in addition to sugar metabolism. The alternation of bacterial compositions and metabolic functions are similar to human T2DM, indicating that T2DM zebrafish can become a suitable alternative model organism to study host-bacterial interaction in human obesity and its-related diseases.

## Supplementary information

Supplementary Materials

Table S1
