# Supplementary material for: Microbiome Alteration in Type 2 Diabetes Mellitus Model of Zebrafish
Source: Sci Rep. 2019 Jan 29;9:867. doi: 10.1038/s41598-018-37242-x (PMC6351536; doi:10.1038/s41598-018-37242-x)
Supplement: Supplementary file 1 — Supplementary Materials [file 41598_2018_37242_MOESM1_ESM.docx]

**Microbiome Alteration in Type 2 Diabetes Mellitus Model of Zebrafish**

Fumiyoshi Okazaki^1-3^*, Liqing Zang^4^, Hiroko Nakayama^4^, Zhen Chen^5^, Zi-Jun Gao^5^, Hitoshi Chiba^6^, Shu-Ping Hui^5^, Takahiko Aoki^1^, Norihiro Nishimura^3,4^, Yasuhito Shimada^2,3,7^*†

^1^ Department of Life Sciences, Graduate School of Bioresources, Mie University, 1577 Kurimamachiya, Tsu, Mie 514-8507, Japan

^2^ Department of Bioinformatics, Mie University Advanced Science Research Promotion Center, Tsu, Mie, Japan

^3^ Mie University Zebrafish Drug Screening Center, Tsu, Mie, Japan

^4^ Graduate School of Regional Innovation Studies, Mie University, Tsu, Mie, Japan

^5^ Faculty of Health Sciences, Hokkaido University, Kita-12, Nishi-5, Kita-ku, Sapporo 060-0812, Japan

^6^ Department of Nutrition, Sapporo University of Health Sciences, Nakanuma Nishi-4-2-1-15, Higashi-ku, Sapporo 007-0894, Japan

^7^ Department of Integrative Pharmacology, Mie University Graduate School of Medicine, Tsu, Mie, Japan

* F. Okazaki and Y. Shimada contributed equally to this work.

† Correspondence: Dr. Yasuhito Shimada, Department of Integrative Pharmacology, Mie University Graduate School of Medicine, 2-174 Edobashi, Tsu Mie, Japan.

Tel: +81 59 231 5411 / FAX: +81 59 232 1765

E-mail: shimada.yasuhito@mie-u.ac.jp

**Supplementary Materials**

**Table S1**

|  | Control | T2DM |
| --- | --- | --- |
| Body weight (g) | 0.31 ± 0.02 | 0.45 ± 0.02^**^ |
| Standard length (mm) | 26.75 ± 0.37 | 29.10 ± 0.18^*^ |
| HAA (mm) | 5.50 ± 0.19 | 6.60 ± 0.22^**^ |
| BMI (kg/m^2^) | 0.28 ± 0.01 | 0.34 ± 0.01^**^ |

**Table S1.** Body metrics after the 4-week feeding experiment. HAA, height at anterior of anal fin. BMI, body mass index. Standard length and HAA were measured as previously described by Parichy DM, *et al.*^1^ and BMI was calculated according to our previous study^2^. **p* < 0.05, ***p* < 0.01, n = 10.

**Figure S1**

**Figure S1.** Tag counts of each sample.

**Figure S2**

**
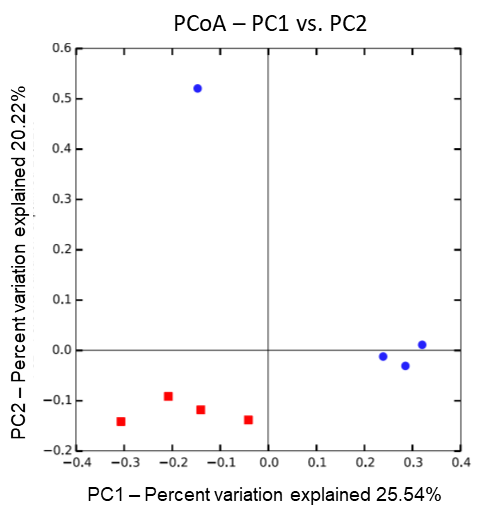
**

**Figure S2.** Principle coordinate analysis (PCoA) of unweighted UniFrac distances. Red and blue spots indicate control and T2DM zebrafish, respectively. Each point represents a different sample, while the red squares and blue circles represent the control and T2DM groups, respectively

**Figure S3**

**
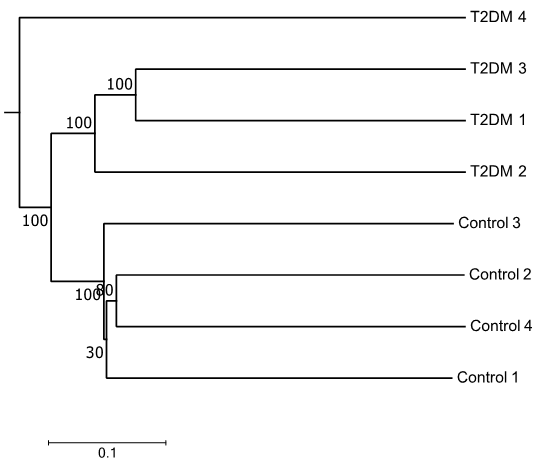
**

**Figure S3.** Jackknifed tree representing the distances between samples, expressed by the unweighted UniFrac metric.

**Figure S4**

**Figure S4.** Bacterial load (CFU/mL) in the experimental tanks. n = 5.

**Figure S5**

**
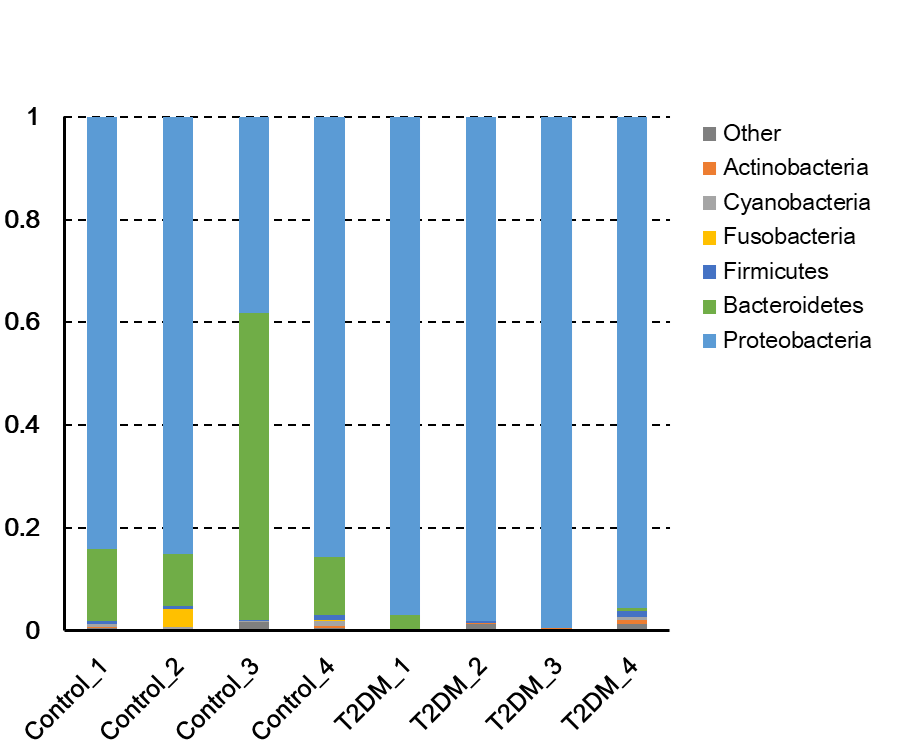
**

**Figure S5.** Bacterial composition at the phylum level.

**Figure S6.**

**
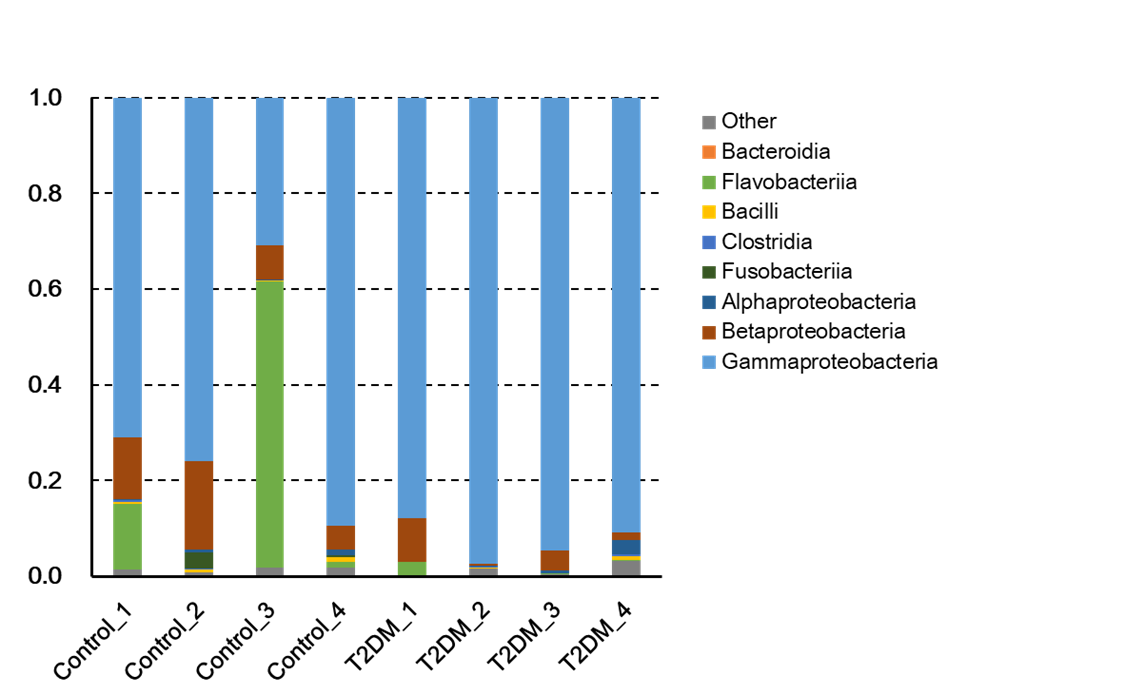
**

**Figure S6.** Bacterial composition at the class level.

**Figure S7**

**Figure S7.** In the Gamma-proteobacteria class, Alteromonadales and Vibrionales showed a decreased in T2DM zebrafish compared to the control group. n = 4.

**Figure S8**

**Figure S8.** In the Bacteroidetes phylum, the Flavobacteria class showed a decreased in T2DM zebrafish compared to the control group. n = 4.

**Figure S9**

**
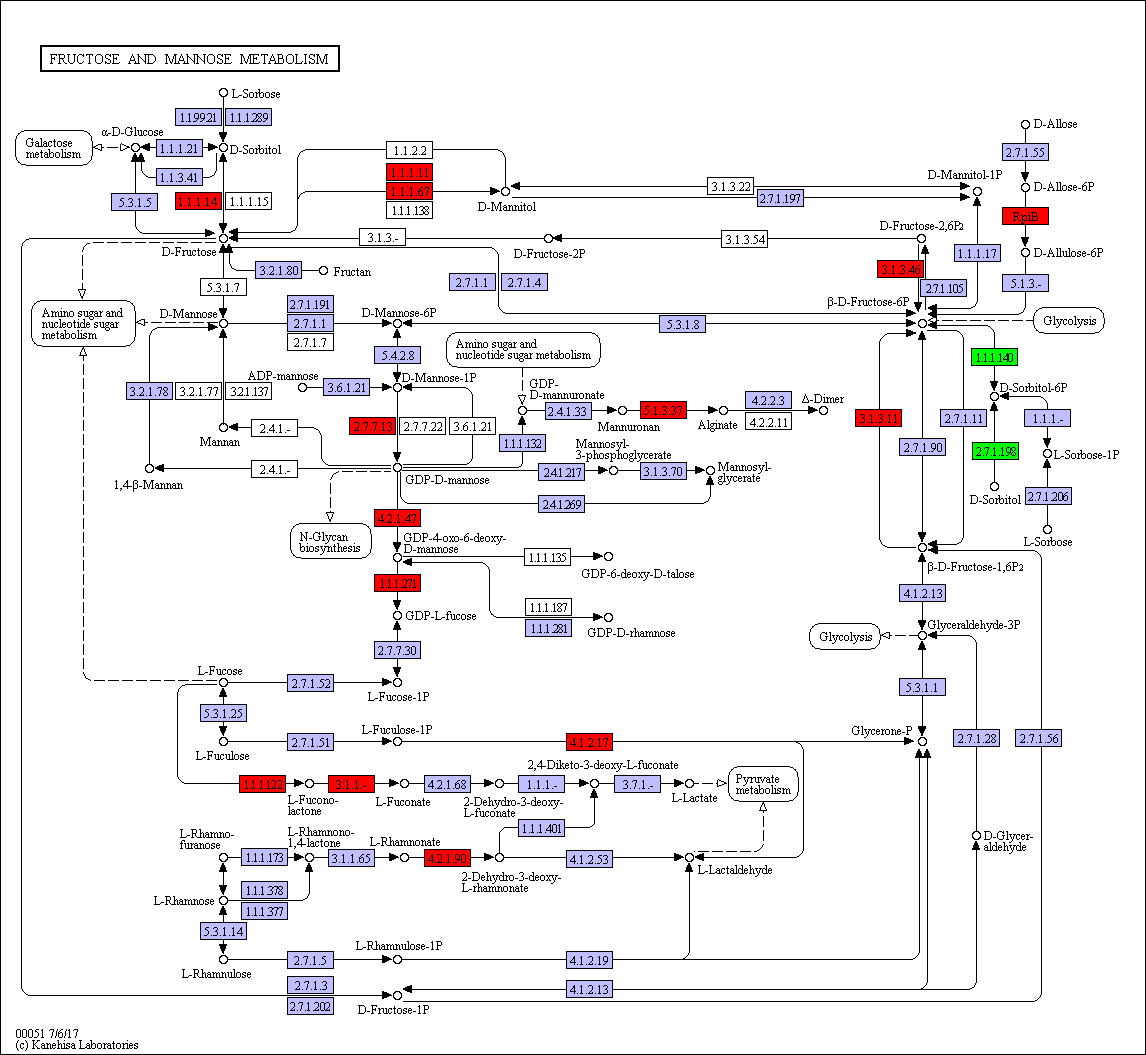
**

**Figure S9.** KEGG pathways involved in fructose and mannose metabolism were dysregulated in T2DM zebrafish compared to the control group. Red and green boxes indicate downregulation and upregulation, respectively. Blue boxes are hyperlinked to KO entries that are selected from the original version in KEGG pathways.

1 Parichy, D. M., Elizondo, M. R., Mills, M. G., Gordon, T. N. & Engeszer, R. E. Normal table of postembryonic zebrafish development: staging by externally visible anatomy of the living fish. *Dev Dyn* **238**, 2975-3015, doi:10.1002/dvdy.22113 (2009).

2 Oka, T. *et al.* Diet-induced obesity in zebrafish shares common pathophysiological pathways with mammalian obesity. *BMC Physiol* **10**, 21, doi:10.1186/1472-6793-10-21 (2010).
